# FUTURE SELF, PERCEIVED CONTROL, AND AGING PREPARATION BEFORE AND AFTER THE PANDEMIC: MODERATED MEDIATION EFFECT

**DOI:** 10.1093/geroni/igac059.2008

**Published:** 2022-12-20

**Authors:** Yue Hu, Zhixuan Lin, Helene Fung

**Affiliations:** The Chinese University of Hong Kong, Hong Kong, Hong Kong; The Chinese University of Hong Kong, Hong Kong, Hong Kong; The Chinese University of Hong Kong, Hong Kong, Hong Kong

## Abstract

Aging preparation, which can be seen as an adaptation to age-related challenges, is always oriented toward the future. The present study aims to explore how thinking about the future self and aging preparation are related. Domain-specific aging preparation was assessed in a longitudinal sample including 359 Hong Kong adults aged from 20 to 98 yrs. In both the pre- (2018) and during pandemic (2020) waves, perceived control fully mediated the positive relationship between thinking clarity on the near future self and aging preparation in the financial domain. However, the mediation effect of perceived control on aging preparation was moderated by age in the pre-pandemic wave, with a higher effect for older adults than for younger adults. Yet, these age differences were no longer significant during the pandemic. The present findings suggested the critical role of perceived control in preparation for the age-related changes and the age-equating effect of COVID-19 pandemic outbreak.

